# iSEE: Interactive SummarizedExperiment Explorer

**DOI:** 10.12688/f1000research.14966.1

**Published:** 2018-06-14

**Authors:** Kevin Rue-Albrecht, Federico Marini, Charlotte Soneson, Aaron T.L. Lun

**Affiliations:** 1 Kennedy Institute of Rheumatology, University of Oxford, Oxford , OX3 7FY, UK; 2 Center for Thrombosis and Hemostasis (CTH), University Medical Center of the Johannes Gutenberg University Mainz, Mainz, Germany; 3 Institute for Medical Biostatistics, Epidemiology and Informatics (IMBEI), University Medical Center of the Johannes Gutenberg University Mainz, Mainz, Germany; 4 SIB Swiss Institute of Bioinformatics, University of Zurich, Zurich, 8057, Switzerland; 5 Institute of Molecular Life Sciences, University of Zurich, Zurich, 8057, Switzerland; 6 Cancer Research UK Cambridge Institute, University of Cambridge, Cambridge, CB2 0RE, UK

**Keywords:** visualization, interactive, R, Bioconductor, genomics, transcriptomics, proteomics, shiny

## Abstract

Data exploration is critical to the comprehension of large biological data sets generated by high-throughput assays such as sequencing. However, most existing tools for interactive visualisation are limited to specific assays or analyses. Here, we present the iSEE (Interactive SummarizedExperiment Explorer) software package, which provides a general visual interface for exploring data in a SummarizedExperiment object. iSEE is directly compatible with many existing R/Bioconductor packages for analysing high-throughput biological data, and provides useful features such as simultaneous examination of (meta)data and analysis results, dynamic linking between plots and code tracking for reproducibility. We demonstrate the utility and flexibility of iSEE by applying it to explore a range of real transcriptomics and proteomics data sets.

## Introduction

Interactive data exploration is critical to the analysis and comprehension of data generated by high-throughput biological assays, such as those commonly used in genomics. Exploration drives the formation of novel data-driven hypotheses prior to a more rigorous statistical analysis, and enables diagnosis of potential problems such as batch effects and low-quality samples. To this end, visualisation of the data using an intuitive and interactive interface is crucial for enabling researchers to examine the data from different perspectives across samples (e.g., experimental replicates, patients, single cells) and features (e.g., genes, transcripts, proteins, genomic regions).

Most existing tools for interactive visualisation of biological data are designed for specific assays and analyses, e.g., pRoloc for proteomics ( Gatto *et al.*, 2014), shinyMethyl for methylation ( Fortin *et al.*, 2014), HTSvis for high-throughput screens ( Scheeder *et al.*, 2017). Opportunities for customisation are generally limited, making it difficult to re-use the same visualisation software for new technologies or experimental designs where different aspects of the data are of interest. Moreover, standalone tools such as the Loupe Cell Browser from 10x Genomics ( Zheng *et al.*, 2017) do not easily integrate into established analysis pipelines such as those based on the R statistical programming language ( R Development Core Team, 2008). This complicates any coordinated use of these tools with a reproducible, transparent, and statistically rigorous analysis.

Here, we present the iSEE software package for interactive data exploration. iSEE is implemented in R using the Shiny framework ( Chang *et al.*, 2017) and exploits data structures from the open-source Bioconductor project ( Gentleman *et al.*, 2004), specifically the SummarizedExperiment class. iSEE allows users to simultaneously visualise multiple aspects of a given data set, including experimental data, metadata and analysis results. Dynamic linking and point selection facilitate the flexible exploration of interactions between different data aspects. Additional functionalities include code tracking, intelligent downsampling of large data sets, custom colour scale specification and tour construction. We demonstrate the capabilities of iSEE by applying it to a diverse range of real data sets.

## Operation

The iSEE software package requires R version 3.5.0 or higher, along with packages from Bioconductor version 3.7 or higher. The interface is initialised with a single call to the iSEE() function, accepting a SummarizedExperiment object ( Huber *et al.*, 2015) as input. Any analysis workflow that generates a SummarizedExperiment object is supported.

## Motivation for using the SummarizedExperiment class

Each instance of the SummarizedExperiment class stores one or more matrices of experimental observations as “assays”, where rows and columns represent genomic features and biological samples, respectively. For instance, individual assays may represent gene expression matrices, either in the form of raw counts or normalised values. In addition, per-feature or per-sample variables are stored in the “rowData” and “colData” slots, respectively; these may include experimental metadata as well as analysis results.

The flexibility of the SummarizedExperiment class is the driving factor behind its broad deployment throughout the Bioconductor ecosystem. SummarizedExperiment objects are currently used in analysis pipelines for RNA sequencing ( Love *et al.*, 2014), methylation ( Aryee *et al.*, 2014) and Hi-C data ( Lun *et al.*, 2016), amongst others. Package developers can also easily use the base SummarizedExperiment class to derive new bespoke classes for particular applications, such as the SingleCellExperiment class for single-cell ‘omics data. By accepting SummarizedExperiment objects as input, iSEE immediately offers interactive visualisation for a variety of data modalities. This complements the state-of-the-art analysis workflows and methodologies already available in R/Bioconductor packages.

## Interface implementation

### Using a multi-panel layout

**Figure 1.**
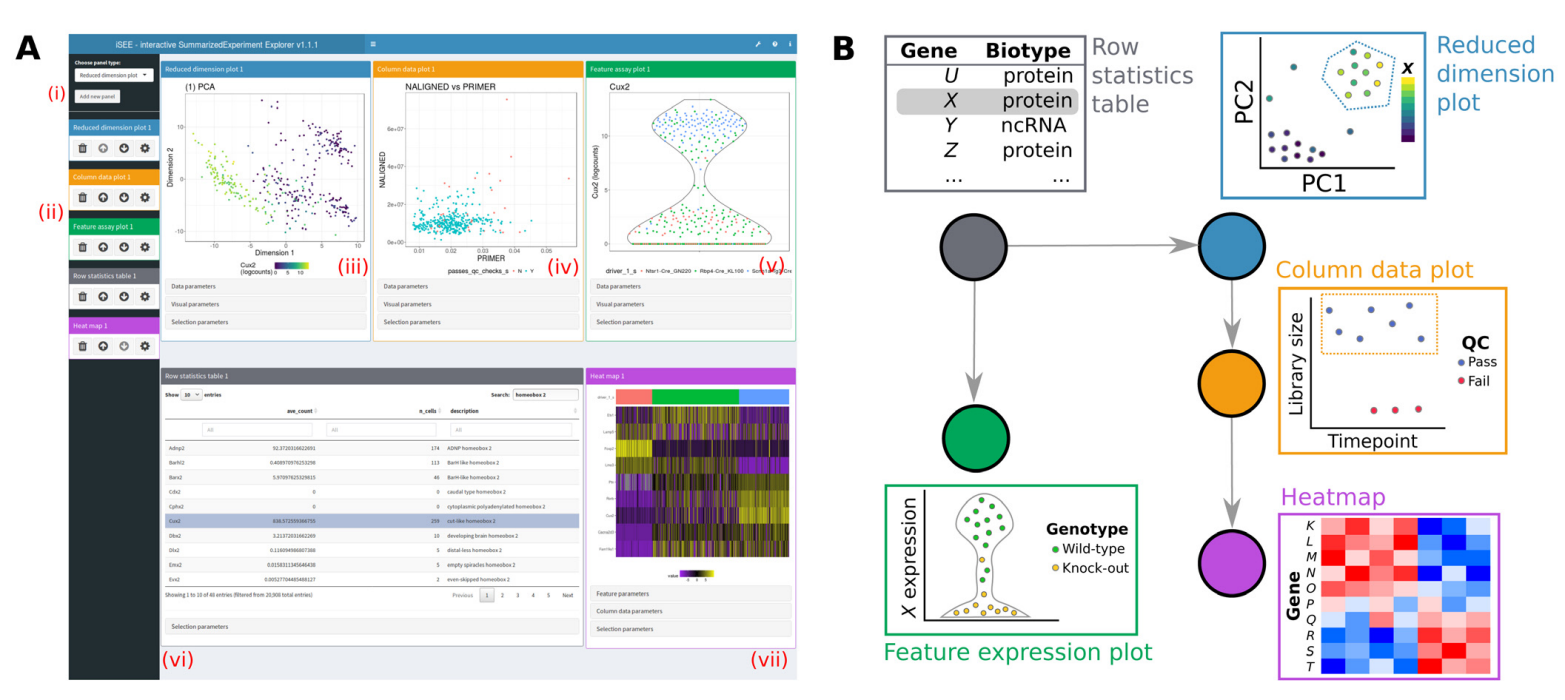
iSEE uses a customisable multi-panel layout ( **A**) that simultaneously displays one or more panels of various types, where each panel type visualises a different aspect of the data. New panels of any type can be added (i), and all panels can be removed, reordered or resized (ii). Panel types are available to visualise sample-based reduced dimensionality embeddings (iii), sample-level metadata (iv), and experimental observations across samples for each feature (v). Other panel types include row statistics tables (vi), to facilitate searching across features and their metadata; heatmaps (vii), to visualise experimental observations for multiple features; and feature-level metadata plots. Panels of each type are colour-coded for ease of interpretation. ( **B**) Information can be transmitted between panels according to a user-specified scheme. Here, the selection of feature *X* in the row statistics table determines the y-axis of the feature assay plot, and colours the samples in the reduced dimension plot by the expression of *X*. Selection of points in the reduced dimension plot (dotted blue line) also determines the samples that are shown in the column data (i.e., sample metadata) plot; further selection of points in the column data plot determines the samples that are shown in the heatmap.

All data aspects stored in a SummarizedExperiment can be simultaneously examined in the multi-panel layout of the iSEE interface ( Figure 1A). The interface layout is built using the shinydashboard package ( Chang & Borges Ribeiro, 2018), with colour-coded panels to visualise each data aspect. Individual panel types include:

- Column data plots, for visualising sample metadata stored in the colData slot of the SummarizedExperiment object.
- Feature assay plots, for visualising experimental observations for a particular feature (e.g. gene) across samples from any assay in the SummarizedExperiment object.
- Row statistics tables, to present the contents of the rowData slot of the SummarizedExperiment object.
- Row data plots, for visualising feature metadata stored in the rowData slot of the SummarizedExperiment object.
- Heatmaps, to visualise assay data for multiple features where samples are ordered by one or more colData fields.
- Reduced dimension plots, which display any two dimensions from pre-computed dimensionality reduction results (e.g., from PCA or *t*-SNE). These results are taken from the reducedDim slot if this is available in the object supplied to iSEE.

Each sample is represented as a point in column data, feature assay and reduced dimension plots. Similarly, each feature is represented by a point in row data plots. For these panel types, a scatter plot is automatically produced if the selected variables on the x- and y-axes are both continuous. If exactly one variable is categorical, points are grouped by the categorical levels and a (vertical or horizontal) violin plot is produced with points scattered within each violin. If both variables are categorical, a “rectangle plot” is produced where each combination of categorical levels is represented by a rectangle with area proportional to the frequency of that combination. Points are scattered randomly within each rectangle. For ease of interpretation, the rectangle plot collapses to a mirrored bar plot when one of the categorical variables only has one level.

### Custom panel colouring

Sample-based points can be coloured according to the values of any sample-level metadata field in the colData slot or by the assay values of a selected feature. Similarly, feature-based points can be coloured according to any feature-level metadata field in the rowData slot. Heatmaps are coloured according to the expression values of the selected features in the chosen assay, with additional colour annotation for each of the colData fields used to order the samples. In all cases, the variable to use for colouring can be dynamically selected for each plot. This enables users to easily examine relationships between different variables in a single plot.

By default, colour maps for categorical and continuous variables are taken from the ggplot2 ( Wickham, 2009) and viridis packages ( Garnier, 2018), respectively. However, iSEE also implements the ExperimentColorMap class, which allows users to specify arbitrary colour maps for particular variables. Each colour map is a function that returns a vector of distinct colours of a specified length, and will be called whenever the associated variable is used for point colouring in a particular panel. The returned colours will be mapped to factor levels for categorical variables, or used in colour interpolation for continuous variables. For categorical variables, the function may also return a constant vector of named colours corresponding to the levels of a known factor. Colour maps can be specified for individual variables; for all assays, all column data variables, or all row data variables (with different functions for continuous or categorical variables); or for all categorical or continuous variables. This provides a convenient yet flexible mechanism for customisation of colouring schemes within the interface.

### Dynamic linking between panels

A key feature of iSEE is the ability to dynamically transmit information between panels ( Figure 1B). Users can define and reorganise arbitrary links between “transmitting” and “receiving” panels, whereby selections in transmitting panels control the inclusion and appearance of the corresponding data points in receiving panels. This feature facilitates exploration of the relationships between different aspects of the data. For example, users can easily determine co-expression patterns of genes in a particular region of a reduced dimensionality embedding – this is achieved by selecting points in a reduced dimension plot (using the standard rectangular brush or a lasso selection) and transmitting that selection to any number of feature assay plots.

This linking paradigm extends to multiple panels, whereby a panel can transmit to multiple receivers, and a receiving panel can transmit its own selection to another plot. Chains of linked plots allow users to mimic the arbitrarily complex gating strategies often found in analyses of flow cytometry data ( Finak *et al.*, 2014). With iSEE, this concept is extended to any assay data, feature-level or sample-level metadata present in a SummarizedExperiment object, providing a powerful framework for interrogating multiple interactions between data aspects. Row statistics tables can also transmit to various plot types, by selecting a table row to control the colouring of sample-based points; or by defining a subset of features to visualise in a heatmap. Furthermore, row data plots can transmit to row statistics tables, whereby selection of points in the former will subset the latter.

### Code tracking and reproducibility

iSEE automatically memorises the exact R code that was used to generate every plot, extending previous work by Marini & Binder (2016). This code is fully accessible to users at any time during the run-time of the interface. By integrating the code reported by iSEE into their own scripts, users can easily reproduce the results of any exploratory analysis. Similarly, the code required to reproduce the current state of the interface can also be reported. This can be used in startup scripts to launch an iSEE instance in any preferred layout, including the panel organisation, variable selection, colouring schemes, links between panels and even individual brushes and lasso selections.

### Additional functionalities

Row statistics tables can be augmented with dynamic annotation based on the selected row, linking to online resources such as Ensembl ( Zerbino *et al.*, 2018) or Entrez ( Coordinators, 2017). For large data sets, points can be downsampled in a density-dependent manner to accelerate rendering of the plots, improving the responsiveness of the interface without compromising the fidelity of the visualisation. Users can also include a bespoke step-by-step “tour” of their data set via the rintrojs package ( Ganz, 2016), guiding the audience through an examination of the salient features in the data.

## Use cases

### Plate-based single-cell RNA sequencing

To demonstrate iSEE’s functionality, we used it to explore a plate-based single-cell RNA sequencing (scRNA-seq) data set involving 379 cells from the mouse visual cortex ( Tasic *et al.*, 2016). This demonstration guides the user through the main features of the iSEE interface including the multi-panel layout, colouring and dynamic linking.

An interactive tour of this use case can be viewed here.

### Droplet-based single-cell RNA sequencing

We applied iSEE to a larger scRNA-seq data set involving 4,000 peripheral blood mononuclear cells (PBMCs), generated by 10x Genomics ( Zheng *et al.*, 2017). This demonstration explores the differences between different methods for distinguishing cells from empty droplets in droplet-based scRNA-seq protocols ( Lun *et al.*, 2018).

An interactive tour of this use case can be viewed here.

### Bulk RNA sequencing from TCGA

We applied iSEE to bulk RNA sequencing data from The Cancer Genome Atlas (TCGA) project, using a subset of expression profiles involving 7,706 tumor samples ( Rahman *et al.*, 2015). This demonstration examines the elevation of *HER2* expression in a subset of breast cancer samples.

An interactive tour of this use case can be viewed here.

### Mass cytometry

Finally, we explored a mass cytometry study involving more than 170,000 PBMCs from multiple donors before and after stimulation with BCR/FcR-XL ( Bodenmiller *et al.*, 2012). We used iSEE to visualise and refine a gating analysis to obtain B cells, and to investigate differences in expression of the functional marker pS6 after stimulation.

An interactive tour of this use case can be viewed here.

## Conclusion

iSEE provides a general interactive interface for visual exploration of high-throughput biological data sets. Any study that can be represented in a SummarizedExperiment object can be used as input, allowing iSEE to accommodate a diverse range of ‘omics data sets. The interface is flexible and can be dynamically customised by the user; supports exploration of interactions between data aspects through colouring and linking between panels; and provides transparency and reproducibility during the interactive analysis, through code tracking and state reporting. The most obvious use of iSEE is that of data exploration for hypothesis generation during the course of a research project. However, we also anticipate that public instances of iSEE will accompany publications to enable authors to showcase important aspects of their data through guided tours.

## Software availability

The iSEE package is available at https://doi.org/doi:10.18129/B9.bioc.iSEE ( Soneson *et al.*, 2018) under an MIT license.

Source code of the development version of the package is available at https://github.com/csoneson/iSEE.

Code for the demonstrations and tours is available at https://github.com/LTLA/iSEE2018.

Archived source code of the version reported in this article and interactive tours is available from http://doi.org/10.5281/zenodo.1247374 ( Rue-Albrecht *et al.*, 2018)

## Data availability

Data used in the described use cases is available from the following articles:

http://doi.org/10.1038/nn.4216 ( Tasic *et al.*, 2016)

http://doi.org/10.1038/ncomms14049 ( Zheng *et al.*, 2017)

https://doi.org/10.1093/bioinformatics/btv377 ( Rahman *et al.*, 2015)

https://doi.org/10.1038/nbt.2317 ( Bodenmiller *et al.*, 2012)
