# Supplementary material for: iSEE: Interactive SummarizedExperiment Explorer
Source: F1000Res. 2018 Jun 14;7:741. [Version 1] doi: 10.12688/f1000research.14966.1 (PMC6013759; doi:10.12688/f1000research.14966.1)
Supplement: Peer review history

# Peer review: Referee response for version 1

**DOI:** 10.5256/f1000research.16293.r35044

**Published:** 2018-06-27

**Authors:** Alejandro Reyes

**Affiliations:** 1 Department of Biostatistics and Computational Biology, Dana-Farber Cancer Institute, Boston, MA, USA

The authors implement an interactive tool, called iSEE, to perform exploratory analyses for high-throughput experiments. The tool inputs a Bioconductor core structure, the SummarizedExperiment object (coerced into a SingleCellExperiment object) and builds an interactive interphase for data exploration. iSEE provides several tools for data exploration by plotting features of an assay along with sample metadata, feature metadata, and reduced representations of the assays. Furthermore, iSEE enables users to interact with the plots and to dynamically link panels with different representations of the data. The analyses performed using iSEE are reproducible, since the code that was run through the graphic interphase can be downloaded.

 Overall, the manuscript presents a very good idea and the code implementation is of great quality. iSEE will be very useful for people without programming background to perform basic analyses. I believe that the success of this tool will depend on whether the authors continue to develop it based on feature requests from users.  

 I don’t have major concerns. However, I do have some recommendations to increase the interest of potential users.

1. Enable users to select more than one group of samples from the dimensionality reduction plots. Furthermore, it would be very useful to enable users to fill new columns of colData based on the interactive grouping of samples.
2. Enable users to retrieve an R data object if the initial input was modified during the analysis.
3. In the context of single-cell or large-scale analyses, it would be helpful to implement tools for differential abundance analyses and gene set enrichment analyses. For instance, one could think of an implementation where users manually define groups of cells from tSNE/PCA plots, retrieve the genes that are differentially expressed between these groups, and extract the pathways that are enriched among the differentially expressed genes.
4. When grouping samples manually on the tSNE/PCA plots, the violin plots of individual features (for example, genes) could be stratified based on these selections (e.g. plot one violin per group of selected points in the “Feature assay plot” panel). In the current implementation, it is only possible to colors the points within the violin plot, which makes difficult to compare distributions between groups of samples.

I have read this submission. I believe that I have an appropriate level of expertise to confirm that it is of an acceptable scientific standard.

# Peer review: Referee response for version 1

**DOI:** 10.5256/f1000research.16293.r35042

**Published:** 2018-06-20

**Authors:** Lorena Pantano

**Affiliations:** 1 Department of Biostatistics, Harvard T. H. Chan School of Public Health, Boston, MA, USA

Authors show an interactive visualization tool for a very common data type used for many of the packages in Bioconductors (SummarizedExperiment). It has enough flexibility to explore all kind of information the object can contain, an interactive tool based on Rshiny, is customizable so it can be adapted to each user.

 I only have minor some comments:

- Tutorial 2: step 10 gets the text box in the upper left of the windows, but I think it should be at other position since it says to change the y-axis of the plot. I think this happens when the user doesn't follow the instruction to click on to some button that should expand the menu with more options.
- It would be nice the tour re-start from the position it was left, with an option to start over. It happened many times that I click accidentally outside the box and I had to start over.
- In the cases the object doesn't have reducedDim for more than the 2 dimensions shown in the plot. I tried to use 3, and it gave an error. Maybe a more informative error would help the user to understand that there is no that information.
- I am not totally sure how to use the rintrojs package to generate a tool. It would be nice a reference to some documentation on how to do it or clarification if I am not understanding this correctly.
- For the features mentioned like code tracking and additional functionality, it would be nice to have a link to the vignette in the paper so the user can jump into how to get it done.
- I think it would be nice to make available a docker image with all the requirements to run iSEE installed. It would promote the use of the tool a lot among bioinformaticians working with non-computational researchers.
- It is nice to change the color for all the variables. I would add an example on how to change the palette for all categorical since the code would be slightly different than the one for continuous variables. It would make the user quickly using that option and avoid silly errors.
- I don't know if this is possible as it is right now, but it could be an option to load a RDA/RDS file containing the SE object instead of creating an app only for that data? That would open the door to deploy the tool independent of the data. For instance, I can see a scenario where iSEE is installed in a docker container, where the user just starts the image and when opening the browser at localhost:8787, there is an option to load a file with the object.

Congrats on the tools!

I have read this submission. I believe that I have an appropriate level of expertise to confirm that it is of an acceptable scientific standard.

# Peer review: Referee response for version 1

**DOI:** 10.5256/f1000research.16293.r35043

**Published:** 2018-06-19

**Authors:** James W. MacDonald

**Affiliations:** 1 Department of Environmental and Occupational Health Sciences, University of Washington, Seattle, WA, USA

The iSEE package was developed to allow people to easily perform exploratory data analysis with data that are stored in a Bioconductor SummarizedExperiment object. A SummarizedExperiment container allows researchers to store one or more matrices of data, where the columns represent samples, and the rows represent either genomic positions or genomic features (genes, exons, transcription start sites, etc). In addition to the matrices of data, the SummarizedExperiment also contains two additional objects that describe the samples (the colData) and the rows (the rowData or rowRanges).

 iSEE allows users to interactively plot the underlying data from a SummarizedExperiment, and also choose subsets of the data based on either interactive selection of data in a plot, or by selecting samples or genomic regions based on the colData or rowData. The chosen subsets can then be linked to other plots in the Shiny Dashboard. This simplifies what could be a complex process, allowing both experienced R users a quick way to check over their data, and allowing less experienced R users the ability to do things that they otherwise might not have been able to do.

 All the underlying code generated while making interactive changes is saved and can be printed out later, in order to make the exploratory data analysis reproducible. This is an excellent feature, particularly for those who want to share observations with colleagues that may not be local. 

 The only negative for this package is that, being based on the Shiny framework, to allow a colleague to explore the data requires that the colleague either have R, iSEE, and all its dependencies installed, or that you have a server running all necessary packages that you can point the colleague to. This limits sharing with people who are not R savvy, but is a function of how Shiny works, rather than the iSEE package.

 This is a high quality package, and given the generalizability of the SummarizedExperiment package, is applicable to a whole range of different data types. Given the ease of use, self documenting features, and applicability to multiple data types, this package will likely become very popular for exploratory data analysis.

I have read this submission. I believe that I have an appropriate level of expertise to confirm that it is of an acceptable scientific standard.
